# Corrigendum to: Clinical spectrum and management of imprinting disorders

**DOI:** 10.1515/medgen-2021-2058

**Published:** 2021-05-14

**Authors:** Miriam Elbracht, Gerhard Binder, Olaf Hiort, Cordula Kiewert, Christian Kratz, Thomas Eggermann

**Affiliations:** Institute of Human Genetics, Medical Faculty, RWTH Aachen University, Pauwelsstr. 30, D-52074Aachen, Germany; Pädiatrische Endokrinologie, Universitätsklinik für Kinder- und Jugendmedizin, Universitätsklinikum Tübingen, Tübingen, Germany; Department of Paediatrics and Adolescent Medicine, Division of Paediatric Endocrinology and Diabetes, University of Lübeck, Lübeck, Germany; Pediatric Endocrinology and Diabetology, Children’s University Hospital Essen, Essen, Germany; Department of Pediatric Hematology and Oncology, Hannover Medical School, Hannover, Germany

The authors wish to correct the legends of tables 1 and 2 as follows:

**Table 1:** Overview on the currently known imprinting disorders, their major molecular findings and main clinical features. (GOM, gain of methylation; IUGR, intrauterine growth restriction; MLID, multilocus imprinting disturbance; LOM, loss of methylation; PNGR, postnatal growth restriction; PTH, parathyroid hormone; upd, uniparental disomy)

**Table 2:** The recently consented clinical diagnostic criteria for SRS, BWS and iPPSDs (4) (5) (6) which might help to decide on the molecular diagnostic testing procedure. For the established diagnostic criteria for PWS and AS we refer to GeneReviews. (PTH parathyroid hormone, TSH thyroid stimulating hormone)

In addition, “parental” was incorrectly named as “paternal” in several places in table 1. A corrected version of table 1 is provided.

**Table 1:** Overview on the currently known imprinting disorders, their major molecular findings and main clinical features. (GOM, gain of methylation; IUGR, intrauterine growth restriction; MLID, multilocus imprinting disturbance; LOM, loss of methylation; PNGR, postnatal growth restriction; PTH, parathyroid hormone; upd, uniparental disomy).

Imprinting disorder OMIM#	Prevalence	Chromosome	Molecular defects (frequency)	Reccurrence risk	MLID	Main clinical features
Transient neonatal diabetes mellitus (TNDM) 601410	1/300,000	6q24	upd(6)pat (41 %)	low		IUGR, transient diabetes mellitus, hyperglycaemia without ketoacidosis, macroglossia, abdominal wall defects
			dup(6q24)pat (33 %)	familial structural variants possible		
			*PLAGL1*:alt-TSS-DMR, LOM (26 %)	in case of MLID: autosomal recessive *ZFP57* mutations	50 %	
Silver–Russell syndrome (SRS) 180860	1/75,000–1/100,000	7	upd(7)mat (7–10 %)	only one familial translocation reported	1 case	IUGR, PNGR, relative macrocephaly at birth, body asymmetry, prominent forehead, feeding difficulties
		11p15.15	upd(11p15)mat (1 case)	not increased		
			dup(11p15)mat (<1 %)	familial structural variants possible		
			*H19/IGF2*:IG:DMR, LOM (40 %)	in case of MLID: maternal effect variants possible (familial) CNVs, SNVs in cis causing LOM reported in rare cases	10 %	
			*CDKN1C*, *IGF2*, *HMGA2*, *PLAG1* point mutations	autosomal-dominant inheritance with parental imprinting		
Birk–Barel syndrome 612292	unknown	8q24.3	*KCNK9* point mutations	autosomal-dominant inheritance with parental imprinting		Intellectual disability, hypotonia, dysmorphism
Beckwith–Wiedemann syndrome (BWS) 130650	1/15,000	11p15.5	upd(11p15)pat (20 %)	no		Macroglossia, exomphalos, lateralized overgrowth, Wilms tumor or nephroblastomatosis, hyperinsulinism, adrenal cortex cytomegaly, placental mesenchymal dysplasia, pancreatic adenomatosis
			dup(11p15)pat (2–4 %)	familial structural variants possible		
			*H19/*IGF2:IG-DMR, GOM (4 %)	OCT4 binding site SNVs in 10 %, might be heritable		
			*KCNQ1OT1*:TSS-DMR, LOM (50 %)	in case of MLID: maternal effect variant	25 %	
			*CDKN1C* point mutations (5 % sporadic; familial 25–50 %)	autosomal-dominant inheritance with parental imprinting		
Temple syndrome (TS14) 616222	unknown	14q32	upd(14)mat (70 %)	familial structural variants possible		IUGR, PNGR, neonatal hypotonia, feeding difficulties in infancy, truncal obesity, scoliosis, precocious puberty, small feet and hands
			del(14q32)pat (10 %)	familial structural variants possible		
			*MEG3/DLK1*:IG-DMR, LOM (20 %)	low	single cases	
Kagami–Ogata syndrome (KOS14) 608149	unknown	14q32	upd(14)pat (70 %)	familial structural variants possible		IUGR, polyhydramnion, abdominal wall defects, bell-shaped thorax, coat-hanger ribs
			del(14q32)mat (20 %)	familial structural variants possible		
			*MEG3/DLK1*:IG-DMR, GOM (10 %)	low		
(familial) Central Precocious Puberty (CPPB)	unknown	14q32	*DLK1* point mutations	autosomal-dominant inheritance with parental imprinting		Central precocious puberty
Prader–Willi syndrome (PWS) 176270	1/25,000–1/10,000	15q11q13	del(15q11q13)pat (70–75 %)	familial structural variants possible		PNGR, Intellectual disability, neonatal hypotonia, hypogenitalism, hypopigmentation, obesity, hyperphagia
			upd(15)mat (25–30 %)	familial structural variants possible		
			*SNURF*:TSS-DMR, GOM (1 %)	0–15 % due to an IC deletion	1 case	
Angelman syndrome (AS) 105830	1/20,000–1/12,000	15q11q13	del(15q11q13)mat (75 %)	familial structural variants possible		Severe intellectual disability, microcephaly, no speech, unmotivated laughing, ataxia, seizures, scoliosis
			upd(15)pat (1–2 %)	familial structural variants possible		
			*SNURF*:TSS-DMR, LOM (1 %)	0–15 % due to an IC deletion		
			*UBE3A* point mutations (10 %)	autosomal-dominant inheritance with parental imprinting		
Central precocious puberty 2 (CPPB2) 615356	Unknown	15q11.2	*MKRN3* point mutations	autosomal-dominant inheritance with parental imprinting		Early activation of the hypothalamic–pituitary–gonadal axis resulting in gonadotropin-dependent precocious puberty
Schaaf–Yang syndrome (SYS) 615547	Unknown	15q11.2	*MAGEL2* point mutations	autosomal-dominant inheritance with parental imprinting		Delayed psychomotor development, intellectual disability, hypotonia
Pseudohypoparathyroidism 1B (PHP1B) 603233	Unknown	20q13	del(20q13)mat (single cases)	familial structural variants possible		Resistance to PTH and other hormones, Albright hereditary osteodystrophy, subcutaneous ossifications, feeding behaviour anomalies, abnormal growth patterns
			*GNAS* DMRs, LOM (>60 %)	familial (structural) variants possible, maternal effect variants in case of MLID	12.5 %	
			upd(20)pat (2–20 %)	familial structural variants possible		
			*GNAS* point mutations	autosomal-dominant inheritance with parental imprinting		
Mulchandani–Bhoj–Conlin syndrome (MBCS) 617352	Unknown	20	upd(20)mat	familial structural variants possible		IUGR, PNGR, feeding difficulties

